# Association of Postoperative Serum Lactate Levels with Acute Kidney Injury in Mexican Patients Undergoing Cardiac Surgery

**DOI:** 10.3390/clinpract14030087

**Published:** 2024-06-07

**Authors:** Héctor-Enrique Flores-Salinas, Anahí de Jesús Zambada-Gamboa, Texali-Candelaria Garcia-Garduño, Guillermo Rodríguez-Zavala, Yeminia Valle, Juan-Carlos Chávez-Herrera, Porfirio-Eduardo Martinez-Gutierrez, Arturo Godinez-Flores, Salvador Jiménez-Limón, Jorge-Ramón Padilla-Gutiérrez

**Affiliations:** 1 Especialidad en Cardiología, Unidad Médica de Alta Especialidad, Centro Médico Nacional de Occidente (CMNO), Departamento de Cardiología, Instituto Mexicano Del Seguro Social (IMSS), Guadalajara 44340, Mexico; 3nriqueflores@gmail.com (H.-E.F.-S.); anahi_cotsin@hotmail.com (A.d.J.Z.-G.); cardiomemo@gmail.com (G.R.-Z.); dr_jcchh@hotmail.com (J.-C.C.-H.); porfirio.martinez@imss.gob.mx (P.-E.M.-G.); arturo_zomy@hotmail.com (A.G.-F.); dr.salvadorjl@hotmail.com (S.J.-L.); 2 Instituto de Investigación en Ciencias Biomédicas (IICB), Centro Universitario de Ciencias de La Salud (CUCS), Universidad de Guadalajara (UDG), Guadalajara 44340, Mexico; texali.garcia3492@alumnos.udg.mx (T.-C.G.-G.); yemivalle@yahoo.com.mx (Y.V.)

**Keywords:** cardiac surgery, kidney injury, lactates, postoperative, risk factors

## Abstract

Acute kidney injury (AKI) is a highly prevalent and a critical complication of cardiac surgery (CS). Serum lactate (sLac) levels have consistently shown an association with morbimortality after CS. We performed a cross-sectional study including 264 adult patients that had a cardiac surgery between January and December 2020. Logistic regression analysis was performed to determine factors associated with AKI development. We measured the postoperative levels of sLac for all participants immediately after CS (T_0_) and at 4 h (T_4_) after the surgical intervention. A linear regression model was used to identify the factors influencing both sLac metrics. We identified four risk predictors of AKI; one was preoperative (atrial fibrillation), one intraoperative (cardiopulmonary bypass time), and two were postoperative (length of hospital stay and postoperative sLac). T_0_ and T_4_ sLac levels were higher among CS-AKI patients than in Non-CS-AKI patients. Postoperative sLac levels were significant independent predictors of CSA-AKI, and sLac levels are influenced by length of hospital stay, the number of transfused packed red blood cells, and the use of furosemide in CS-AKI patients. These findings may facilitate the earlier identification of patients susceptible to AKI after CS.

## 1. Introduction

Acute kidney injury (AKI) is a clinical syndrome that is characterized by an abrupt alteration in kidney function, as indicated by an increase in one’s serum creatinine (sCr) level and/or a reduction in urine output [1]. AKI represents a common postoperative complication in patients undergoing cardiac surgery (CS) and percutaneous coronary interventions with prolonged intensive care unit (ICU) stays [2,3,4]. Cardiac-surgery associated AKI (CS-AKI) leads to increased mortality rates in the postoperative stage [5], and this augmented mortality remains high for 10 years after CS, even in patients with complete renal recovery [6]. There is a complex interaction between patient and procedure-related factors that may contribute to the development of CS-AKI, and these factors can be grouped into preoperative, intraoperative, and postoperative factors [7].

Regarding the first group, the preoperative variables comprise demographic characteristics such advanced age and sex, but they also include preexisting kidney alterations, obesity, hypertension, diabetes mellitus, cardiac dysfunction, sepsis, volume depletion, hepatic failure, and exposure to nephrotoxic substances, including drugs [7]. The intraoperative factors comprise hypovolemia, hypotension, kidney ischemia, inflammation, transfusion, decreased cardiac output, the use of diuretics, vasopressors and inotropes, and the use of cardiopulmonary bypass (CPB). Finally, the postoperative factors encompass hypovolemia, reduced cardiac output, mechanical ventilation, the consumption of potentially nephrotoxic drugs, and urinary obstruction [7,8].

Recently, the use of biomarkers associated with hypoperfusion and ischemia has been proposed as a clinical tool for the prediction of CS-AKI. In this context, serum lactate (sLac) is directly associated with capillary perfusion, and it is a microcirculation biomarker [9,10,11].

Due to its physiologic interrelation with the kidneys and the production of lactate, this biochemical parameter could be established as a screening tool for identifying and monitoring patients with developing CS-AKI. Also, there are several reports indicating an association between sLac levels and morbidity and mortality after CS [12,13]. Therefore, we aimed to identify the risk factors related to CS-AKI development and to analyze the association of postoperative sLac levels with CS-AKI in Mexican patients.

## 2. Materials and Methods

We performed a cross-sectional study and included individuals admitted to the postsurgical unit of the Western National Medical Center of the Mexican Institute of Social Security (Jalisco, México) between January and December 2020. All eligible patients were chosen by consulting the patients’ postsurgery records. AKI was defined using the current KDIGO (Kidney Disease: Improving Global Outcomes) 2012 guideline [1], which includes the following: an increase in SCr by ≥0.3 mg/dL within 48 h; or an increase in SCr to ≥1.5 times baseline within the previous 7 days; or urine volume < 0.5 mL/kg/h for 6 h. A total of 264 expedients of adult patients (≥18 years of age) that had underwent cardiac surgery were included; 66 patients had a postsurgical diagnosis of AKI, and 198 did not. Considering a prevalence of AKI of 23% [14], the proportion of patients diagnosed with cardiac surgery-associated acute kidney injury (CS-AKI) with respect to the patients that did not develop CS-AKI (Non-CS-AKI) was 3:1, and the participants were grouped by age. Patients with Chronic Kidney Disease (CKD) or patients who were discharged or died after postsurgical unit admission were excluded from the study. 

The analyzed variables were obtained from individual handwritten clinical registers and encompassed demographic variables (sex and age); the presence of comorbidities (diabetes mellitus, smoking, hypertension, obesity, atrial fibrillation, left ventricular ejection fraction, and dyslipidemia); the presence of infection; the preoperative level of sCr; the preoperative administration of angiotensin-converting enzyme inhibitors (ACEIs), angiotensin-II receptor antagonists (ARAs), and furosemide; postsurgical transfusion; CPB time; and the postsurgical use of antiarrhythmics. Aortic clamp time was not included in our regression analysis due to its high collinearity with CPB time. The postoperative levels of sLac in whole blood were measured postoperatively at time zero (T_0_) after CS and were also quantified at 4 h (T_4_) after CS using an amperometric metabolite sensor. The machine used for lactate measurement was a GEM Premier 3000 with its own cartridges and GEM IQM cartridges (Werfen, Bedford, MA, USA). To strengthen the interpretation of our findings, we validated our results by comparing them with established normal reference ranges and data from healthy individuals.

The device uses the amount of electrical current flowing through the sensor inside the device to calculate the concentration of lactate being oxidized

Categorical variables were described as the total number and percentage for each category. Continuous variables were indicated as the median and [interquartile range]. The Mann–Whitney U test was used to analyze continuous variables that do not fit a normal distribution. A chi-square test was used to compare the categorical variables. Univariate and multivariate logistic regression analyses were performed to identify risk factors for AKI. Only variables with statistical significance in the univariate analysis were integrated in the multivariate analysis model (enter model). Data were described as odds ratios (ORs) with 95% confidence intervals (CIs). Statistical significance was established at *p*-value < 0.05. Statistical analysis was performed with the statistical software package GraphPad Prism (version 9.5.1).

## 3. Results

### 3.1. Clinical Characteristics

The clinical and demographic characteristics of the studied groups are detailed in Table 1. The mean age was 60 and 59 years in the CS-AKI and Non-CS-AKI groups, respectively (*p* = 0.4407). The proportion of males was higher in both groups. The prevalence of obesity and atrial fibrillation were significantly higher in the CS-AKI group. Eight AKI patients (12%) underwent renal replacement therapy (RRT), and four individuals with AKI (6%) had hemodialysis. CPB time was greater in patients that had developed AKI compared to patients who did not (*p* = 0.0001). Patients diagnosed with AKI were more likely to require antiarrhythmic and vasopressor drugs (*p* = 0.004 and *p* = 0.001, respectively) as compared to the Non-CS-AKI patients. In the same way, the hospital length of stay was longer in patients who had developed AKI when compared to the Non-CS-AKI participants (*p* < 0.0001). The use of furosemide was more prevalent in patients who had not developed AKI when compared to the CS-AKI group (*p* < 0.0001). As expected, the mean value of sCr was statistically different between the groups (0.88 ± 0.25 CS-AKI and 1.54 ± 7.7 Non-CS-AKI, *p* = 0.0091).

The types of surgery and their frequencies (presented as percentage for the CS-AKI group and that for the Non-CS-AKI group, respectively) in the CS-AKI and Non-CS-AKI groups were as follows: coronary artery bypass surgery (41% and 35%), aortic valve replacement (18% and 20%), replacement of two or more valves (17% and 6%), mitral valve replacement (7.5 and 6%), coronary artery bypass surgery and mitral valve replacement (6% and 3.5%), ascending aortic surgery (6% and 5%), mediastinal exploration (1.5% and 4.5%), and others (3% and 20%).

### 3.2. Serum Lactate Levels

**Figure 1:**
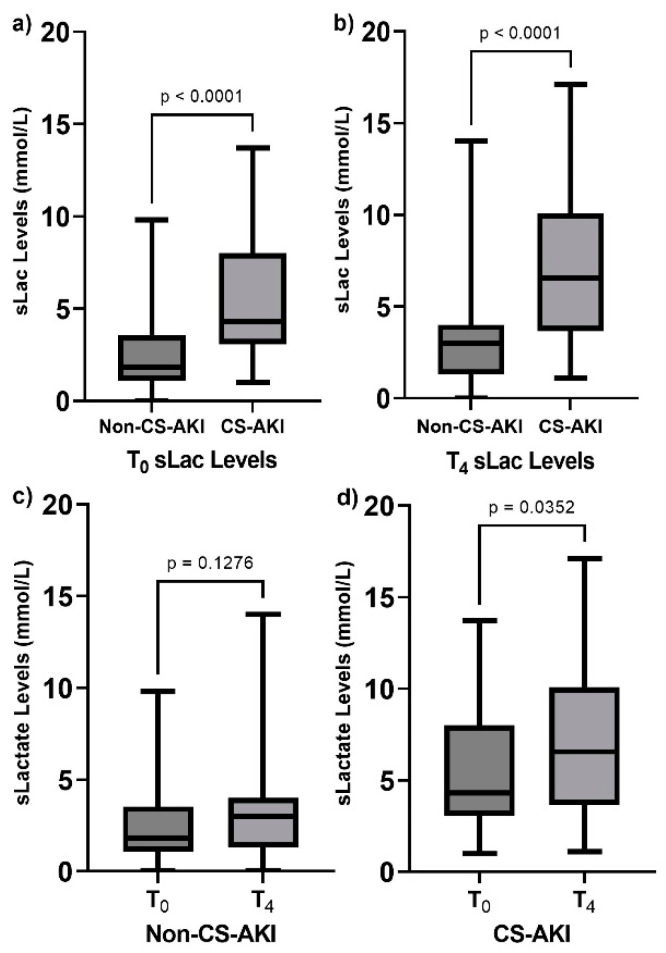
Comparison of postoperative serum lactate (sLac) levels in the studied groups. (**a**) sLac levels at T_0_ in both groups, (**b**) sLac levels at T_4_ in both groups, (**c**) comparison of sLac levels in the Non-CS-AKI group at T_0_ vs. T_4_, and (**d**) comparison of sLac levels in the CS-AKI group at T_0_ vs. T_4_. Data are presented as median and interquartile range. Data were analyzed through the use of the Mann–Whitney U test.

Regarding T_0_ sLac levels, the values were higher among the CS-AKI individuals as compared with the Non-CS-AKI individuals (4.3 [3.2–7.77] mmol/L vs. 2.6 [2.0–2.85] mmol/L, *p* < 0.0001) (Figure 1a). In the same way, the sLac levels at T_4_ showed an increase in patients who had developed AKI compared to those who had not (6.55 [3.75–10.0] mmol/L vs. 3.0 [2.0–4.0] mmol/L, *p* < 0.0001) (Figure 1b). Also, when we compared the levels of sLac at T_0_ and at T_4_ in each group, the CS-AKI group showed an increase in sLac levels (4.3 mmol/L vs. 6.5 mmol/L, *p* = 0.04), as presented in Figure 1d, and this was not observed in the Non-CS-AKI patients (Figure 1c).

### 3.3. Multivariate Analysis

Furthermore, we evaluated the factors that influence the level of T_0_ and T_4_ sLac thorough a linear regression analysis. The variables significantly associated with T_0_ and T_4_ sLac are indicated in Table 2.

Additionally, according to the multivariate analysis, the occurrence of AKI was influenced by sex, hospital length of stay, both postoperative times for the quantification of sLac levels, the use of furosemide, and the presence of atrial fibrillation. The statistical parameters for the logistic regression model are indicated in Table 3. As the presence of obesity and atrial fibrillation influenced the risk of CS-AKI, we analyzed the levels of sLac in these subsets of patients. When comparing the level of sLac at T_0_ in CS-AKI individuals with obesity (n = 34, 4.85 [3.60–8.22] mmol/L) and in the Non-CS-AKI individuals with obesity (n = 68, 2.4 [1.8–3.3] mmol/L), there was a significant difference (*p* < 0.0001). In the same subsets, the levels of T_4_ sLac in the CS-AKI and Non-CS-AKI individuals were 6.0 [3.77–10.00] mmol/L and 3.0 [2.0–4.0] mmol/L, respectively. Concerning the presence of atrial fibrillation, there were significant differences when comparing the level of T_0_ sLac in the subset of the CS-AKI group (n = 24, 5.0 [2.97–10.00] mmol/L) and the subset of the Non-CS-AKI group (n = 34, 2.0 [1.7–3.0] mmol/L) with the presence of this risk factor (*p* < 0.0001). Moreover, when adjusting for independent variables, the levels of both sLac level measurement times were not predicted by the presence of obesity or atrial fibrillation (Table 3).

### 3.4. Receiver Operating Characteristic Curve of Serum Lactate Levels

**Figure 2:**
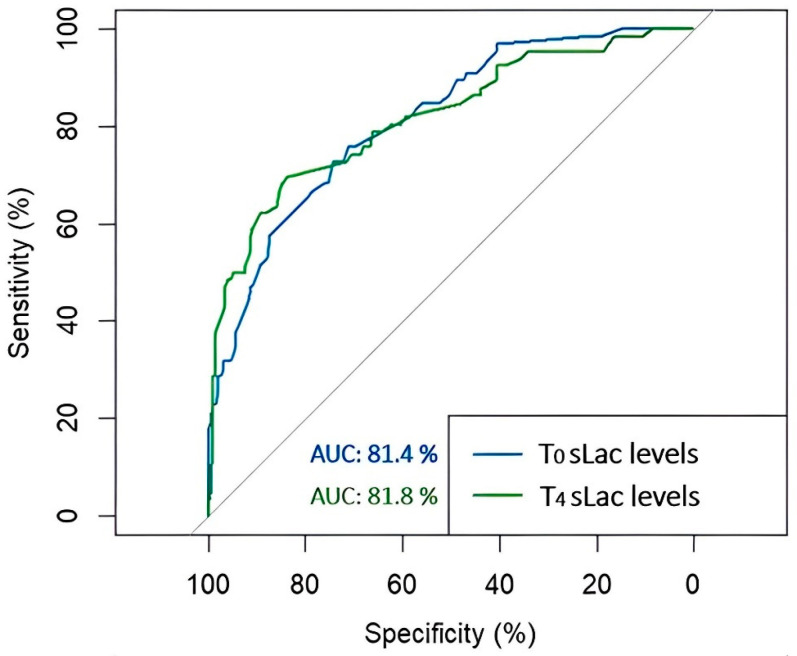
Receiver operating characteristic (ROC) curves for the development of acute kidney injury after cardiac surgery according to the levels of T_0_ and T_4_ serum lactate (sLac). The area under the ROC curve was 0.814 for T_0_ sLac (blue), and it was 0.818 for T_4_ sLac (green).

Finally, to estimate the predictive accuracy of the T_0_ and T_4_ sLac levels for the development of CS-AKI, receiver operating characteristic analysis was performed (Figure 2). The area under the curve (AUC) of T_0_ sLac was 0.814 (95% CI: 0.756–0.872), and for T_4_ sLac, it was 0.818 (95% CI: 0.755–0.882). The optimum cut-off points and the values of sensitivity–specificity were 3.45 mmol/L (72–74%) for T_0_ sLac and 4.05 mmol/L (70–83%) for T_4_ sLac. According to the sLac level, based on the optimal cut-off values, we observed that 72.7% of patients (n = 48) had a T_0_ sLac level ≥ 3.45 mmol/L, and 73.7% of individuals in the Non-CS-AKI group (n = 146) had a sLac level < 3.45 mmol/L. Concerning T_4_ sLac level, 69.7% (n = 46) of patients in the CS-AKI group had a sLac level ≥ 4.05 mmol/L, and 80.3% (n = 158) showed a sLac concentration < 4.05 mmol/L.

## 4. Discussion

The incidence and mortality of AKI has been increasing during the past few decades, even though there have been advances in critical care and treatment technologies. The actual assumption about its physiopathological mechanisms is that hypoperfusion to the kidney, because of decreased cardiac output, decreased mean arterial pressure, or both, are the main causes of injury in patients undergoing CS [6]. Also, it has been pointed out that a complex interaction between patient and procedure-related factors contribute to the development of AKI after CS [15,16].

Concerning the preoperative risk factors, the male sex was predominant compared to women in both groups (*p* = 0.04). There are controversies regarding the role of sex in the pathophysiology, prevalence, and progression of renal disorders. Some reports suggest that females present an elevated tendency to develop AKI compared to men [2,6], but reports also exist suggesting that female sex is a protective factor in renal disease [17]. In concordance with the latest reports, in the present study, as indicated by the multivariate analysis, the female sex was identified as a protective factor in the development of AKI after CS (OR: 0.24, 95% CI: 0.07–0.68, *p* = 0.011). In this setting, the female sex’s protective association with the development of CS-AKI may be related to the protective effects of estrogen, as it has been reported that this type of hormone decreases renal oxidative stress [18].

Moreover, obesity was more frequent in the CS-AKI group when compared to the Non-CS-AKI group (51.5% vs. 34%, *p* = 0.001), and in the same way, atrial fibrillation was more prevalent in the CS-AKI group than in the Non-CS-AKI group (36% vs. 17%, *p* = 0.001). However, after adjustment, only atrial fibrillation was independently associated with the development AKI in patients undergoing CS (OR: 3.11, 95% CI: 1.08–9.40, *p* = 0.037). In concordance with this finding, Wang et al. reported that the risk of in-hospital AKI increased significantly in patients hospitalized due to atrial fibrillation [19]. Furthermore, the presence of risk factors for atrial fibrillation often coexists with and may predispose to the development of kidney disease, and vice versa. However, it is worth noting that AKI itself is linked to a higher mortality; so, the coexistence of these two diseases significantly reduces the life expectancy of affected individuals. For this reason, understanding this pathophysiologic interaction requires further investigation.

Regarding the intraoperative factors, in the multivariate analysis, we identified that CPB time was higher in the CS patients that had developed AKI compared to the patients who had not developed AKI (*p* < 0.0001 and *p* = 0.0009, respectively), and this association has been consistent among several reports [5,20,21]. The deleterious effect of CPB on kidney function is multifactorial, and it is now well established that a long CPB time is considered as a potential modifiable risk factor [20].

In addition, concerning the drugs that were part of the intraoperative and postoperative stages, patients diagnosed with CS-AKI were more likely to require antiarrhythmic and vasopressor drugs (*p* = 0.004 and *p* = 0.001, respectively) when compared to the Non-CS-AKI individuals. On the other hand, the use of furosemide was more frequent in the Non-CS-AKI group than in patients who developed AKI (*p* < 0.0001). Though, only the administration of furosemide influenced the risk of AKI after adjusting in the corresponding model. In fact, the administration of diuretics has a controversial association with the risk of developing AKI after CS. On the one hand, according to retrospective studies on patients undergoing CS, the use of furosemide may lead to hypercreatinemia [22], and it has been associated with worse renal function [23]. On the other hand, the use of furosemide has been suggested to have a nephroprotective effect [5]. In studies including healthy subjects, the diuresis effect is produced by 10 mg of furosemide, with its maximal effect occurring at a dose of 40 mg when it is administered intravenously [24]. In the present investigation, the furosemide dose ranged from 10 to 100 mg, and we observed that at ≤40 mg, the use of furosemide had a protective impact on the risk of CS-AKI (OR: 0.09, 95% CI: 0.02–0.36, *p* = 0.001), as indicated by the multivariate analysis. In this context, the variability among reports may reflect, in part, the variable dosing that is usually employed based on disease severity. 

Several reports have supported the use of sLac concentration as a measure of inadequate perfusion and tissue hypoxia, and this biochemical parameter has been proposed as a screening tool for identifying patients with underlying tissue hypoperfusion, and it could be used to distinguish critically ill patients from less critically ill individuals [25]. Nevertheless, it is currently not possible to define the best time interval between lactate measurements [26]; so, a dynamic assessment of serial lactate levels may be more informative than a single measurement. According to a systematic review, lactate quantification every 1–2 h is probably sufficient in most acute conditions [26]. According to the present study, in the logistic regression analysis, both postoperative measurement times for sLac were independent risk factors for CS-AKI development. Previously, Zhang et al. reported that postoperative normalized lactate load was independently associated with postoperative AKI in patients undergoing CPB [27]. Additionally, in the particular setting of CS-AKI, Radovic et al. evaluated NGAL (Neutrophil Gelatinase-Associated Lipocalin), KIM-1 (Kidney Injury Molecule-1), and sLac levels in CS patients assessed as low-risk for developing CS-AKI, and they found out that postoperative sLac was a better predictor of CS-AKI than NGAL and KIM-1 [28]. It is worth mentioning that in this study, the researchers included a relatively small number of CS-AKI patients (n = 15), and they evaluated only low-risk CS-AKI patients and measured lactate levels in arterial blood samples.

Additionally, we evaluated the factors influencing T_0_ and T_4_ sLac levels. We found out that transfusion, the use of furosemide, and the length of hospital stay influenced the concentrations of sLac. In the present study, the median number of transfused PRBCs was five and three units in the CS-AKI and Non-CS-AKI groups, respectively. Surgenor et al. studied the use of ≤3 PRBC units to reflect the management of routine intraoperative anemia during CS, and the transfusion of >3 RBCP represents the management of active hemorrhage or severe anemia [29]. As the number of PRBCs ranged from 0 to 10, we grouped patients receiving ≤3 units and individuals who received more than 4 units of PRBCs. In the linear regression analysis, we observed that the use of ≤3 units was negatively associated with the levels of sLac at two times of quantification (T_0_ β = −1.16, *p* < 0.0001; T_4_ β = −1.64, *p* < 0.0001, respectively). The transfusion of PRBC units is used to improve oxygen delivery (DO_2_) and increase the proportion of global DO_2_ and global oxygen consumption (VO_2_). Lactate is the most commonly available surrogate of the DO_2_–VO_2_ balance, and it has been proposed as a PRBC transfusion trigger [26]. In this context, if hemoglobin decreases to such an extent that the demand for tissue DO_2_ can no longer be met, tissue hypoxia occurs, resulting in a rise in lactate levels [30]. In terms of our study’s observations, the use of ≤3 PRBC units was negatively associated with postoperative levels of sLac, and this may reflect the improvement in the DO_2_–VO_2_ balance during CS due to transfusion when the number of PRBCs was ≤3.

Moreover, according to our linear regression analysis, when furosemide was not administered, the level of sLac decreased (T_0_ sLac: −1.31, *p* = 0.003; T_4_ sLac: −2.76, *p* < 0.0001). However, when furosemide was used at a dose ≤ 40 mg, it also had a negative effect on the levels of sLac, and in concordance with the result of the logistic regression model, the use of such a dose of furosemide had a protective effect on the risk of CS-AKI, as mentioned above. Considering that the administration of furosemide before and after CS is a method for decreasing the risk of fluid overload [6,31] and that fluid overload has been reported as an independent risk factor for AKI [32], in our cohort, the association between furosemide use and sLac levels may suggest that there is a relationship between fluid balance and sLac levels. However, further investigations regarding this association in CS-AKI patients need to be carried out.

Finally, in the current study, the length of stay in the postsurgical unit was longer in patients who had developed AKI when compared to the Non-CS-AKI individuals (5 vs. 3 days, *p* < 0.0001, respectively). Several studies have reported this observation. In addition, in patients with CS-AKI, a longer length of stay in the ICU, as well as in hospital, was associated with an increased risk of death [5,6,7,33,34]. Also, the hospital length of stay influenced the levels of sLac. In accordance with these observations, Andersen et al. reported that increased postoperative sLac levels were associated with increased hospital length of stay for patients undergoing major CS [11]. Given the fact that sLac levels have been shown to be independently associated with the risk of CS-AKI and that there is a relationship between sLac concentration and length of hospital stay, which, in turn, is associated with high morbidity and mortality [35], this points out that the underlying mechanisms of lactate increase could be a promising target for further investigation and that they could be used as an intervention target to improve patient life expectancy.

The findings of the present study should be understood in the context of the study’s design and limitations. As the data were collected retrospectively, the investigation was limited by the variables collected; so, potential confounding due to unreported variables and the multiple other possible causes of raised sLac levels may be present in the patients’ postsurgery results. Also, because of the study’s design, we could not determine the exact mechanism of the complex interplay between CS-AKI, lactate levels, and other well-documented risk factors. As well as this, the current study had a relatively short observational period, and for this reason, we were unable to perform a statistical analysis related to the association between sLac levels and mortality. Preoperative sLac levels were not available, and we were unable to adjust for this variable. Nevertheless, Radovic et al. reported that postoperative sLac was a better predictor of CS-AKI risk than preoperative sLac values [28]. Regarding the use of furosemide, we do not have a precise chronology of the time of its administration. Also, we did not obtain information about urinary output when adjusting for furosemide’s effect on renal function. Also, the single-center design potentially affects the generalizability of the findings. Therefore, the results may not be applicable to other settings or populations, and this should be considered when interpreting the study’s implications. However, the remaining hospitals of the Mexican Social Security Institute use the same perioperative protocols for cardiac surgery, so the external validity could be extrapolated to these centers. Additionally, the small sample sizes for the subset-specific comparisons present a significant limitation in terms of statistical power. Still, this study has a larger sample size compared to other similar studies. For these reasons, our observations need to be confirmed by additional studies with larger sample sizes.

Furthermore, this study highlights a critical need for detailed data collection on transfusions and underlying diseases. Comprehensive records on the number and type of blood transfusions, as well as detailed medical histories, are essential for a thorough analysis and understanding of the factors influencing serum lactate levels and the development of CS-AKI. Additionally, this study underscores the importance of extensive serum lactate data collection over time. Monitoring serum lactate levels at more frequent intervals could provide better insights into dynamic changes and the potential predictive value of lactate for CS-AKI. This would enable a more nuanced understanding of the relationship between lactate levels and postoperative outcomes.

## 5. Conclusions

Postoperative sLac levels were significant independent predictors of CSA-AKI, and sLac levels are influenced by length of hospital stay, the number of transfused PRBCs, and the use of furosemide in CS-AKI patients. These findings may facilitate the earlier identification of patients susceptible to AKI after CS.

## Figures and Tables

**Table 1:** Baseline characteristics of cardiac surgery-associated acute kidney injury (CS-AKI) and without Acute Kidney Disease (Non-CS-AKI) individuals according to the categories of risk factors by surgical stage.

Variable	CS-AKI *n* = 66 (%)	Non-CS-AKI *n* = 198 (%)	*p*-Value
Preoperative risk factors			
Age	60	59	0.4407
Gender			
Female	12 (18.2)	64 (32.3)	0.04
Male	54 (81.8)	134 (67.7)	
T2DM	26 (39.4)	53 (26.8)	0.07
Smoking	26 (39.4)	71 (35.8)	0.71
Systemic Arterial Hypertension	42 (63.6)	103 (52)	0.13
Obesity	34 (51.5)	68 (34.3)	0.019
Dyslipidemia	16 (24.2)	52 (26.3)	0.870
Diastolic dysfunction	54 (81.8)	136 (68.6)	0.057
Atrial fibrillation	24 (36.3)	34 (17.2)	0.002
ACEI consumption	17 (25.7)	38 (19.2)	0.33
ARA consumption	26 (39.4)	56 (28.3)	0.12
Intraoperative risk factors			
Transfusion	66 (100)	185 (93.4)	NA
<3 PRBCs	28 (43)	145 (73)	NA
≥4 PRBCs	37 (56)	53 (27)	NA
CPB time	156.0 [124.2–191.5]	121.5 [87.25–159.75]	<0.0001
AxC time	109 [88–150]	90 [67.5–116.8]	0.0009
Use of inotropes	54 (81.8)	142 (71.7)	0.14
Postoperative risk factors			
Use of Furosemide	15 (22.7)	178 (89.8)	<0.0001
Antiarrhythmics	23 (34.8)	34 (17.2)	0.004
Use of Vasopressors	56 (84.8)	123 (62.1)	0.001
Hospital length of stay (days)	5 [3–8]	3 [2–5]	<0.0001

Quantitative data are presented as median [interquartile range], tested by the Mann–Whitney U test; proportions are indicated as total number (percentage), analyzed by a chi-square test. Abbreviations: T2DM: Type 2 Diabetes Mellitus; ACEIs: angiotensin-converting enzyme inhibitors; ARA: angiotensin-II receptor antagonist; PRBCs: packed red blood cells; CPB: cardiopulmonary bypass; and AxC: aortic cross clamp.

**Table 2:** Linear regression analysis of factors influencing postoperative levels of serum lactate.

Variable	T_0_ Serum Lactate Level			T_4_ Serum Lactate Level		
	β	OR [95%CI]	*p*-Value	β	OR [95%CI]	*p*-Value
Hospital length of stay	0.11	1.11 [1.05, 1.17]	0.0002	0.08	1.10 [1.03–1.18]	0.009
Transfusion						
≤3 PRBCs	−1.16	0.31 [0.18, 0.53]	<0.0001	−1.64	0.17 [0.08, 0.34]	<0.0001
Furosemide use						
≤40 mg	−0.95	0.38 [0.16, 0.89]	0.027	−2.20	0.12 [0.04, 0.36]	<0.0001

T_0_ serum lactate-adjusted R-squared model: 0.33, *p* < 0.0001; T_4_ serum lactate-adjusted R-squared model: 0.34, *p* < 0.0001. Abbreviations: PRBCs: packed red blood cells; T_0_: quantification of serum lactate immediately after cardiac surgery; T_4_: quantification of serum lactate 4 h after cardiac surgery.

**Table 3:** Logistic regression analysis of factors influencing the risk of cardiac-surgery associated acute kidney injury.

Variable	β Coefficient	OR [95% CI]	*p*-Value
Female gender	−1.46	0.23 [0.06, 0.71]	0.015
Hospital length of stay	0.13	1.15 [1.04, 1.29]	0.013
CPB time	0.02	1.03 [1.01–1.05]	0.008
Atrial fibrillation	1.13	3.11 [1.08–9.40]	0.037
T_0_ serum lactate	0.36	1.44 [1.12, 1.91]	0.007
T_4_ serum lactate	0.31	1.38 [1.13, 1.69]	0.001
Use of furosemide ≤ 40 mg	−2.52	0.08 [0.02, 0.31]	0.0005

Abbreviations: CPB: cardiopulmonary bypass, T_0_: quantification of serum lactate immediately after cardiac surgery, T_4_: quantification of serum lactate 4 h after cardiac surgery.

## Data Availability

The raw data supporting the conclusions of this article will be made available by the authors on request.

## References

[1] Kellum J.A., Lameire N. (2013). Diagnosis, evaluation, and management of acute kidney injury: A KDIGO summary (Part 1). Crit. Care.

[2] Harky A., Joshi M., Gupta S., Teoh W.Y., Gatta F., Snosi M. (2020). Acute Kidney Injury Associated with Cardiac Surgery: A Comprehensive Literature Review. Braz. J. Cardiovasc. Surg..

[3] Pickkers P., Darmon M., Hoste E., Joannidis M., Legrand M., Ostermann M., Prowle J.R., Schneider A., Schetz M. (2021). Acute kidney injury in the critically ill: An updated review on pathophysiology and management. Intensive Care Med..

[4] Su L.J., Li Y.M., Kellum J.A., Peng Z.Y. (2018). Predictive value of cell cycle arrest biomarkers for cardiac surgery-associated acute kidney injury: A meta-analysis. Br. J. Anaesth..

[5] Thiele R.H., Isbell J.M., Rosner M.H. (2015). AKI associated with cardiac surgery. Clin. J. Am. Soc. Nephrol..

[6] Wang Y., Bellomo R. (2017). Cardiac surgery-associated acute kidney injury: Risk factors, pathophysiology and treatment. Nat. Rev. Nephrol..

[7] Boyer N., Eldridge J., Prowle J.R., Forni L.G. (2022). Postoperative Acute Kidney Injury. Clin. J. Am. Soc. Nephrol..

[8] Hobson C., Ruchi R., Bihorac A. (2017). Perioperative Acute Kidney Injury: Risk Factors and Predictive Strategies. Crit. Care Clin..

[9] Gonçalves M., Gameiro J., Pereira M., Rodrigues N., Godinho I., Neves M., Gouveia J., Jorge S., Lopes J.A. (2017). Serum lactates and acute kidney injury in patients with sepsis: A cohort analysis. Cogent Med..

[10] Yan G., Wang D., Tang C., Ma G. (2021). The Association of Serum Lactate Level with the Occurrence of Contrast-Induced Acute Kidney Injury and Long-Term Prognosis in Patients Undergoing Emergency Percutaneous Coronary Intervention. Int. J. Gen. Med..

[11] Gomez-Martinez R., Tlacuilo-Parra A., Garibaldi-Covarrubias R. (2007). Use of complementary and alternative medicine in children with cancer in Occidental, Mexico. Pediatr. Blood Cancer.

[12] Andersen L.W., Holmberg M.J., Doherty M., Khabbaz K., Lerner A., Berg K.M., Donnino M.W. (2015). Postoperative Lactate Levels and Hospital Length of Stay After Cardiac Surgery. J. Cardiothorac. Vasc. Anesth..

[13] El-Khoury J.M., Hoenig M.P., Jones G.R.D., Lamb E.J., Parikh C.R., Tolan N.V., Wilson F.P. (2021). AACC Guidance Document on Laboratory Investigation of Acute Kidney Injury. J. Appl. Lab. Med..

[14] Chavez-Iniguez J.S., Madero M. (2022). Global Perspectives in Acute Kidney Injury: Mexico. Kidney360.

[15] Kellum J.A., Romagnani P., Ashuntantang G., Ronco C., Zarbock A., Hans-Joachim A. (2021). Acute kidney injury. Nat. Rev. Dis. Primers.

[16] Negi S., Koreeda D., Kobayashi S., Iwashita Y., Shigematu T. (2016). Renal replacement therapy for acute kidney injury. Ren. Replace. Ther..

[17] Bairey-Merz C.N., Dember L.M., Ingelfinger J.R., Vinson A., Neugarten J., Sandberg K.L., Sullivan J.C., Maric-Bilkan C., Rankin T.L., Kimmel P.L. (2019). Sex and the kidneys: Current understanding and research opportunities. Nat. Rev. Nephrol..

[18] Darvishzadeh-Mahani F., Khaksari M., Raji-Amirhasani A. (2021). Renoprotective effects of estrogen on acute kidney injury: The role of SIRT1. Int. Urol. Nephrol..

[19] Wang G., Yang L., Ye N., Bian W., Ma C., Zhao D., Liu J., Hao Y., Yang N., Cheng H. (2021). In-hospital acute kidney injury and atrial fibrillation: Incidence, risk factors, and outcome. Ren. Fail..

[20] Karim H.M., Yunus M., Saikia M.K., Kalita J.P., Mandal M. (2017). Incidence and progression of cardiac surgery-associated acute kidney injury and its relationship with bypass and cross clamp time. Ann. Card. Anaesth..

[21] López-Delgado J.C., Esteve F., Torrado H., Rodríguez-Castro D., Carrio M.L., Farrero E., Javierre C., Ventura J.L., Manez R. (2013). Influence of acute kidney injury on short- and long-term outcomes in patients undergoing cardiac surgery: Risk factors and prognostic value of a modified RIFLE classification. Crit. Care.

[22] Lombardi R., Ferreiro A., Servetto C. (2003). Renal function after cardiac surgery: Adverse effect of furosemide. Ren. Fail..

[23] Lassnigg A., Donner E., Grubhofer G., Presterl E., Druml W., Hiesmayr M. (2000). Lack of renoprotective effects of dopamine and furosemide during cardiac surgery. J. Am. Soc. Nephrol..

[24] Matsuura R., Komaru Y., Miyamoto Y., Yoshida T., Yoshimoto K., Isshiki R., Mayumi K., Yamashita T., Hamasaki Y., Nangaku M. (2018). Response to different furosemide doses predicts AKI progression in ICU patients with elevated plasma NGAL levels. Ann. Intensive Care.

[25] Okorie O.N., Dellinger P. (2011). Lactate: Biomarker and potential therapeutic target. Crit. Care Clin..

[26] Vincent J.L., Quintairos E.S.A., Couto L., Taccone F.S. (2016). The value of blood lactate kinetics in critically ill patients: A systematic review. Crit. Care.

[27] Zhang Z., Ni H. (2015). Normalized lactate load is associated with development of acute kidney injury in patients who underwent cardiopulmonary bypass surgery. PLoS ONE.

[28] Radovic M., Bojic S., Kotur-Stevuljevic J., Lezaic V., Milicic B., Velinovic M., Karan R., Simic-Ogrizovic S. (2019). Serum Lactate as Reliable Biomarker of Acute Kidney Injury in Low-risk Cardiac Surgery Patients. J. Med. Biochem..

[29] Surgenor S.D., DeFoe G.R., Fillinger M.P., Likosky D.S., Groom R.C., Clark C., Helm R.E., Kramer R.S., Leavitt B.J., Klemperer J.D. (2006). Intraoperative red blood cell transfusion during coronary artery bypass graft surgery increases the risk of postoperative low-output heart failure. Circ. J..

[30] Czempik P.F., Gierczak D., Wilczek D., Krzych Ł.J. (2022). The Impact of Red Blood Cell Transfusion on Blood Lactate in Non-Bleeding Critically Ill Patients-A Retrospective Cohort Study. J. Clin. Med..

[31] Fakhari S., Bavil F.M., Bilehjani E., Abolhasani S., Mirinazhad M., Naghipour B. (2017). Prophylactic furosemide infusion decreasing early major postoperative renal dysfunction in on-pump adult cardiac surgery: A randomized clinical trial. Res. Rep. Urol..

[32] Wang N., Jiang L., Zhu B., Wen Y., Xi X.M. (2015). Fluid balance and mortality in critically ill patients with acute kidney injury: A multicenter prospective epidemiological study. Crit. Care.

[33] Hobson C., Ozrazgat-Baslanti T., Kuxhausen A., Thottakkara P., Efron P.A., Moore F.A., Moldawer L.L., Segal M.S., Bihorac A. (2015). Cost and Mortality Associated with Postoperative Acute Kidney Injury. Ann. Surg..

[34] Mandelbaum T., Scott D.J., Lee J., Mark R.G., Malhotra A., Waikar S.S., Howell M.D., Talmor D. (2011). Outcome of critically ill patients with acute kidney injury using the Acute Kidney Injury Network criteria. Crit. Care Med..

[35] Mak N.T., Iqbal S., de Varennes B., Khwaja K. (2016). Outcomes of post-cardiac surgery patients with persistent hyperlactatemia in the intensive care unit: A matched cohort study. J. Cardiothorac. Surg..

